# Vaccine decision making in New Zealand: a discrete choice experiment

**DOI:** 10.1186/s12889-024-17865-8

**Published:** 2024-02-12

**Authors:** Amy Hai Yan Chan, Marvin Tao, Samantha Marsh, Helen Petousis-Harris

**Affiliations:** 1 School of Pharmacy, University of Auckland, Level 3, Building 505, 85 Park Road, Grafton, 1023 Auckland, New Zealand; 2 School of Medicine, University of Auckland, Building 505, 85 Park Road, Grafton, 1023 Auckland, New Zealand; 3 School of Population Health, University of Auckland, 85 Park Road, Grafton, 1023 Auckland, New Zealand

**Keywords:** Vaccination, Discrete choice, Decision making, Attributes, Preferences

## Abstract

**Background:**

Vaccine hesitancy is a significant threat to global health. A key part of addressing hesitancy is to ensure that public health messaging prioritises information that is considered important to the public. This study aimed to examine how different vaccine characteristics affect public preferences for vaccines in New Zealand, what trade-offs they are willing to make between different vaccine characteristics, and how their preferences are affected by their vaccine-related conspiracy beliefs and COVID-19 vaccination status.

**Methods:**

An online discrete choice experiment (DCE) was designed to elicit individual preferences about vaccines using the 1000minds platform. Members of the general population of New Zealand aged ≥ 18 years were invited to complete the DCE. Participants were asked to indicate their preference between two options showing different combinations of vaccine characteristics. Data on sociodemographic characteristics were collected. Beliefs were measured using the vaccine conspiracy beliefs scale (VCBS) with scores ≥ 19 indicating strong vaccine-related conspiracy beliefs. The DCE was analysed using the PAPRIKA method (Potentially All Pairwise RanKings of all possible Alternatives) and preferences compared between respondents with high versus low VCBS scores and vaccinated versus unvaccinated respondents for COVID-19.

**Results:**

A total of 611 respondents from 15 regions completed the DCE. Mean (SD) age was 45.9 (14.7) years with most having had 2 or more doses of the coronavirus vaccine (86%). Mean (SD) VCBS score was 18.5 (12.4) indicating moderate vaccine-related conspiracy beliefs. Risk of severe adverse effects was the most highly valued vaccine characteristic, followed by vaccine effectiveness and duration of protection. Vaccine origin and route of administration were ranked least important. Respondents scoring high on the VCBS placed less value on the effectiveness of vaccines but greater value on development time and total number of doses (*p* < 0.001). COVID-19 unvaccinated respondents ranked development time and total number of doses more highly than those vaccinated respondents (*p* < 0.001).

**Conclusions:**

Risk of severe adverse effects, vaccine effectiveness and duration of protection were rated by the New Zealand public as the top three most important vaccine characteristics. This information is important for informing public health messaging to promote vaccine uptake and inform vaccine decision-making.

**Supplementary Information:**

The online version contains supplementary material available at 10.1186/s12889-024-17865-8.

## Background

Despite the abundance of evidence supporting vaccines and immunisation programmes, hesitancy to receive vaccines remains common and is a significant public health issue [1–4]. Vaccine hesitancy is listed as one of the top ten significant threats to global health by the World Health Organization (WHO) [5]. This hesitancy has undermined the efforts and achievements from vaccines [6], and has been exacerbated by the coronavirus disease 2019 (COVID-19) pandemic [7–10], with studies showing that many people have been, and remain, hesitant about COVID-19 vaccination [7, 9, 11]. Vaccine hesitancy has far-reaching effects on public health, with outbreaks of vaccine-preventable illnesses such as measles [12–14], pertussis [15, 16], and poliomyelitis [17] occurring in different parts of the world, due to inadequate vaccination rates.

A key part of addressing vaccine hesitancy is to ensure that public health messaging about vaccines addresses the concerns of individuals and prioritises information that is considered important to them. Several large meta-analyses have shown that the decision an individual makes about whether or not to take a medication is influenced by people’s beliefs about the treatment– their perceived need for the medication versus any concerns the individual may have about side effects and harm. These beliefs are affected by the individual’s medicines information needs and preferences [18–21]. To promote vaccine uptake, it is therefore important to identify exactly what information different communities want and need about their vaccines, so that the information health providers and governments give about vaccines meets the public’s information needs.

A method that has been increasingly used in medical decision-making research to explore individual preferences about healthcare is a discrete choice experiment (DCE). A DCE is a quantitative method that can elicit individual preferences by asking respondents to make a choice from two or more scenarios without directly asking them to state their preferred options. DCEs have long been established as a sound and valid method of identifying the preferences of groups of people. With roots in economics and marketing, DCEs have been used to elicit the preferences in consumers for products and services such as clean-fuel vehicles [22], and hotel rooms [23]. DCEs have now been increasingly used in studies concerning healthcare [24–27], and specifically to study people’s preferences for vaccines [28–30]. DCEs provide information on how important different attributes of an item, such as a vaccine, are to an individual by considering what is most important according to the person’s preference.

Previous DCE studies of vaccine preferences conducted overseas have found that ‘vaccine effectiveness’ was the most important attribute [28, 31, 32]. The rankings of other attributes varied; however, attributes such as ‘the risk of adverse effects’ and ‘duration of protection’ were also shown to be important [28, 33]. Other studies have found vaccine safety and availability of the vaccine by walk-in or mobile clinics to be important attributes [34]. How this translates to New Zealand is not currently known as there is no prior literature examining the vaccine preferences of people in New Zealand and how different sociodemographic characteristics may influence their preferences [28].

The aim of the study was to explore, using a DCE, the New Zealand public’s preferences for information about different vaccine attributes, what trade-offs they are willing to make between different attributes, and how their preferences are affected by their vaccine-related conspiracy beliefs and COVID-19 vaccination status. Specifically, the study aims to address several research questions: (1) what vaccine attributes are most important to New Zealanders when making decisions about vaccination; (2) how do the different vaccine attributes rank in terms of importance; (3) how do individuals’ vaccine preferences vary between people with high versus low vaccine-related conspiracy beliefs; and (4) how do individuals’ vaccine preferences differ between people who have received the COVID-19 vaccine versus those who have not?

This study makes a unique contribution to the literature by addressing these research gaps. The findings from this study can inform public health physicians, health professionals and policymakers which vaccine attributes are most important to New Zealanders when it comes to vaccine decision-making and therefore inform tailoring of patient information accordingly. The findings also lay a foundation for the development and evaluation of vaccine promotion interventions.

## Methods

To address the first two research questions, we used a DCE methodology to identify which vaccine attributes are most important, and the order of importance:

### Designing the Discrete Choice experiment (DCE)

**Fig. 1:**
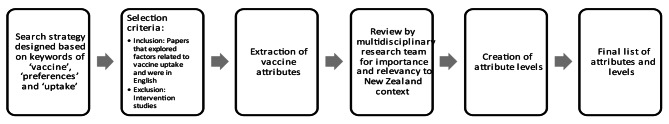
Literature review process to develop DCE attribute and levels

The first step of designing a DCE is selecting what attributes and levels should be included. A literature review was conducted to identify vaccine attributes and levels that can influence people’s preferences for vaccines, an approach that has been adopted by other DCEs of vaccine preferences [35–37](see Fig. 1 for details of the literature review process and Appendix A1 for search terms used).

PubMed, EMBASE and Google Scholar was searched using terms related to vaccines, immunisation, patient preferences, attitudes and uptake (see Appendix A1 for search terms). The approach was adapted from Diks et al. [28] and Dong et al. [35]. Studies that explored factors related to vaccine uptake and were in English were included. No limit was placed in relation to the type of vaccines as this DCE relates to general vaccination. Intervention studies were excluded as this would modify the relationship between identified variables and the outcome (vaccine uptake). Due to the large volume of existing qualitative work already completed both within and outside New Zealand on factors relating to vaccine uptake [38–43], we did not conduct a further qualitative research phase. The first DCE version comprised the following seven attributes which were identified from the literature: vaccine effectiveness, duration of protection, adverse effects, number of injections, country of origin, route of administration and time taken to develop the vaccine. A further two attributes (frequency of injections and vaccine activation period) were added after initial review of the retrieved attributes with the research team and based on clinical experience from the group. The list of attributes and levels were then reviewed by a multidisciplinary team of researchers, comprising representatives from vaccinology, population health, pharmacy and medicine, to determine the final attributes and levels. Attributes were reviewed by the team, and either included or excluded from the final DCE depending on whether expert consensus review based on their expertise and evaluation of the attributes. Consensus agreement had to be reached for attributes to be removed or added and disagreements were resolved by consensus discussion within the group. From the feedback, the attributes frequency and vaccine activation period were removed, but a further 5 attributes were added: out-of-pocket cost, burden of disease, accessibility, local and population coverage. These attributes were changed based on expert consensus review from the multidisciplinary team, as it was felt that ‘Frequency’ overlapped with ‘Number of total doses required’, and ‘Vaccine activation period’ was too variable between individuals and vaccine to be included. Further detail on the rationale for adding and removing attributes are described in Appendix A2. The levels were further refined and changes to the wording of the DCE were made to improve relevancy and understanding. Appendix A2 summarises the iterative changes made to the DCE attributes and levels. Those that were included in our DCE were developed following the criteria of Hensher et al. [44]. The criteria ensures their relevance and efficacy by checking (a) that all levels and their combinations are plausible; (b) the levels and their combinations are familiar to the participants; and (c) the variability of the levels are considered in the design to ensure the participants can make trade-offs between them. The final attributes and levels were confirmed by expert consensus and only variables that were considered realistic and relatable to New Zealand were considered. This was so that the DCE results could be applicable to the New Zealand setting– for example, in New Zealand, vaccines are generally funded so an out-of-pocket cost of $0 is possible, which may not be the case in other DCEs.

Table 1 shows the final set of 13 attributes and levels. The attributes were identified from literature relating to a range of vaccines including childhood vaccinations, influenza, human papillomaviruses (HPV), meningococcal B, varicella, and hypothetical vaccines such as human immunodeficiency virus (HIV). These included vaccine effectiveness [35, 37, 45–51], duration of protection [35, 45, 46, 48–50], risk of mild adverse effects [45, 49, 51], risk of severe adverse effects [45, 48, 51, 52], total number of doses [35, 37, 50], place of origin [35, 46], route of administration [36, 37], out-of-pocket costs [35–37, 46, 48–51], burden of disease [36, 46, 47], accessibility [47, 48, 51], local coverage [47], and population coverage [47, 49, 51]. In addition to these attributes, a new attribute, vaccine development time, was included based on advice from the research team given the initial concerns in the global community about the speed of development of the coronavirus vaccines. The final set was decided upon after consensus discussion with the multidisciplinary team of researchers. The set comprised a large number of attributes and levels, more so than may be expected in a usual DCE. This is because each of these were deemed to be important factors in the literature and relevant for the New Zealand population by the research team, and secondly, the PAPRIKA DCE method chosen accommodates a larger set of attributes and levels as it is a type of adaptive DCE. The PAPRIKA approach serves to minimise the number of questions a respondent is asked while ensuring all possible attributes are reviewed (see section below for further information) [53].

**Table 1:** Attributes and levels for general vaccines for the discrete choice experiment

Attributes	Levels
Vaccine effectiveness [35, 37, 45–51]	50%
	70%
	90%
Duration of protection [35, 45, 46, 48–50]	1 year
	10 years
	Lifetime
Risk of mild adverse effects (e.g. cold, fever, muscle aches) [45, 49, 51]	1 in 50
	1 in 20
	1 in 10
Risk of severe adverse effects (e.g. allergic reactions) [45, 48, 51, 52]	1 in 1,000,000
	1 in 100,000
	1 in 1,000
Total number of doses [35, 37, 50]	One
	Two
	Three
Place of origin [35, 46]	New Zealand
	Asia
	Europe
	USA
	Multiple countries
Route of administration [36, 37]	Needle injection into muscle
	Skin patch
	Oral (by mouth)
	Nasal spray
Development time	1 year
	5 years
	10 + years
Out-of-pocket cost [35–37, 46, 48–51]	$0
	$5
	$50
	$100–150
Burden of disease [36, 46, 47]	Common with mild symptoms. Hospitalisations are rare and the disease is not life-threatening.
	Common with severe symptoms. Hospitalisations are common and the disease is life-threatening.
	Rare with mild symptoms. Hospitalisations are rare and the disease is not life-threatening.
	Rare with severe symptoms. Hospitalisations are common and the disease is life-threatening.
Accessibility [47, 48, 51]	Community-based healthcare (e.g. GPs, pharmacies, drive-in vaccine centres, community centres)
	Workplaces and schools
	Hospitals
Local coverage [47]	20% of your family and friends are already vaccinated
	50% of your family and friends are already vaccinated
	80% of your family and friends are already vaccinated
Population coverage [47, 49, 51]	20% of the population is already vaccinated
	50% of the population is already vaccinated
	80% of the population is already vaccinated

### Designing DCE trade-offs and scenarios

**Fig. 2:**
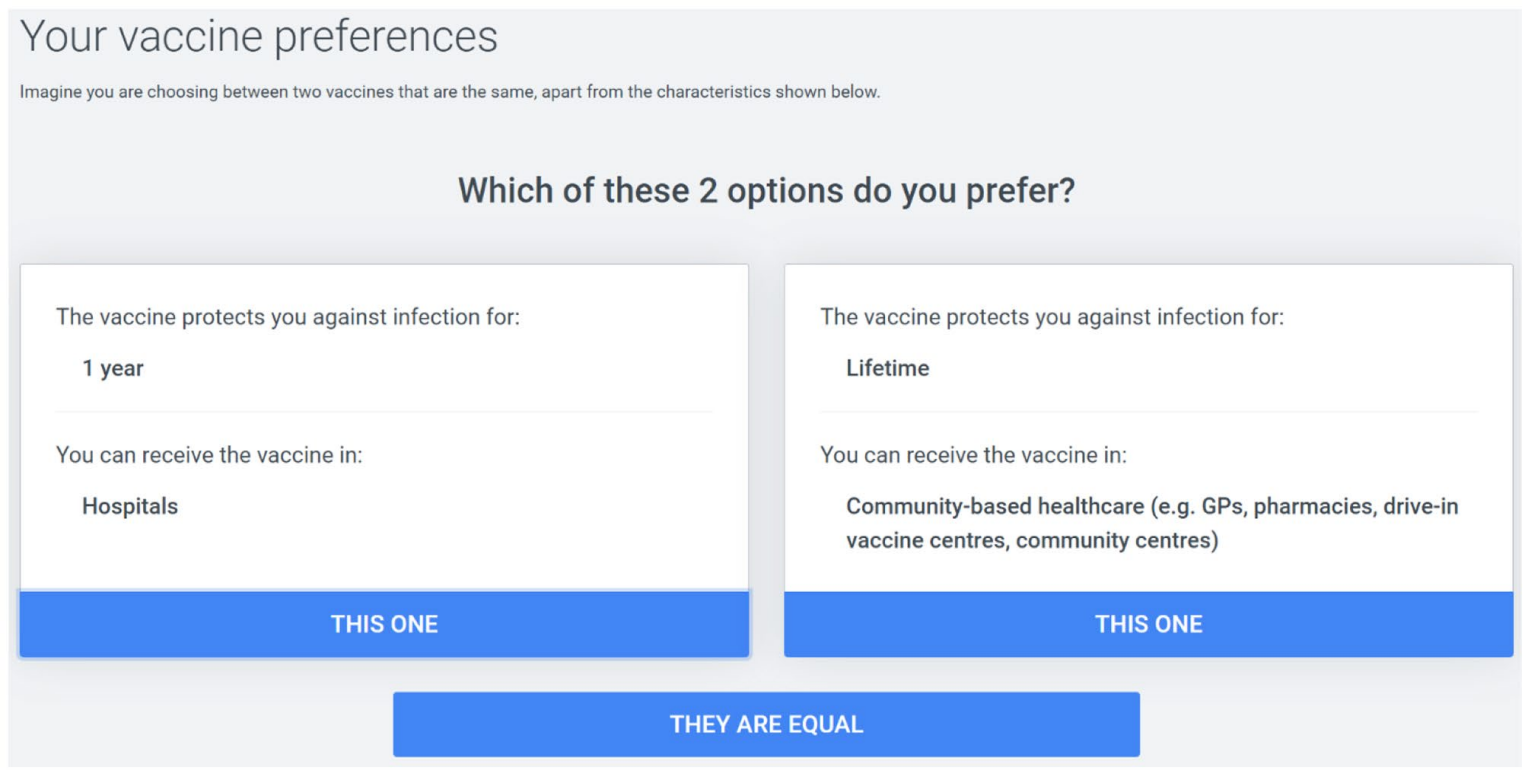
Example of discrete choice question from the 1000minds software

This DCE utilised the PAPRIKA method — Potentially All Pairwise RanKings of all possible Alternatives [54]. This method of conducting DCEs has been previously demonstrated to be effective in studies concerning health applications, such as in asthma treatment [55], health technology prioritisation [56–58], and prioritisation of patients for elective surgery [59]. The method involves presenting participants with two hypothetical vaccines with differences in only two attributes, with the other attributes remaining identical. An example of a question is depicted in Fig. 2. Participants were asked to indicate their preference— a ‘trade-off’ — between two options showing different combinations of vaccine characteristics. Participants were also able to indicate indifference between the two options by choosing “They are equal” as a response. This approach is a partial profile type of DCE as the alternatives within the choice set is defined on the two attributes, with the other attributes missing from the choice set being treated as being the same. This approach was chosen due to the large number of attributes which would appear under a full-profile DCE regimen, where there would be multiple attributes the respondent would need to review. The use of simple choice sets means the comparisons are relatively easy to think about. Partial-profile conjoint analysis has been shown to reflect participants’ true preferences more accurately than full-profile conjoint analysis [60, 61].

A series of these scenarios were presented randomly to capture the participants’ preferences for each attribute combination. The PAPRIKA method is considered an adaptive DCE, as each of the participants’ answers to a choice set will influence the following set that they will be presented. A non-adaptive method would involve the same group of choice sets being presented to each participant, however this is often not practical because the number of possible sets increases exponentially with the number of attributes and levels. In contrast, as PAPRIKA is an adaptive method, the choice sets that participants end up answering are determined in real time as they progress through them. For example, to minimise the number of choices that participants must make, the PAPRIKA method implements the ‘law of transitive property’ (transitivity), where the software identifies sets of vaccine alternatives that the participant has already shown a preference for or against and prevents them from appearing again in future questions. This ensures that subsequent questions will always involve choice sets whose answers cannot be implied, either explicitly or implicitly by transitivity, by the previous responses, thus reducing redundancy and participant burden. This transitivity can be illustrated in this example: if a person prefers Vaccine X to Vaccine Y, but then states they prefer Vaccine Y to Vaccine Z, then by transitivity, Vaccine X is also preferred to Vaccine Z, so this comparison is not asked about. The number of choice sets presented to each participant will vary between participants depending on their previous responses. If participants opt out of choice sets repeatedly, they will still be shown the set eventually at a later point in the DCE, unless they leave the DCE questionnaire early.

### DCE configuration

The final DCE comprised three sections. The first section required the participants to rank, from ‘highest’ to ‘lowest’, levels of attributes that could not be objectively ranked by the researchers as no obvious ranking from most to least preferable exists. For example, attributes such as place of origin, route of administration, and accessibility have levels that do not have an obvious ‘preferable answer’. For these attributes, participants were asked to rank the levels themselves prior to the completing the choice tasks. The second section displayed the DCE trade-offs between the different attributes and levels described previously.

To address the second two research questions, we collected data on participants’ vaccine conspiracy beliefs and COVID-19 vaccination status in the third section of the DCE. To measure vaccine conspiracy beliefs, we used the validated vaccine conspiracy beliefs scale (VCBS) [62, 63]. The VCBS scale measures the degree that participants endorse conspiracy theories about vaccines [64] and identifies extreme anti-vaccination views. It consists of seven statements and requires participants to state to what extent do they agree with these statements using a seven-point scale — strongly disagree (1) to strongly agree (7). This has been shown to predict COVID-19 vaccine hesitancy [62, 63]. Higher scores indicate that the participant was more likely to endorse vaccine-related conspiracies. Scores < 19 were regarded as low scores (i.e. minimal beliefs in vaccine-related conspiracies) and ≥ 19 as high scores (i.e. strong beliefs in vaccine-related conspiracies), a threshold score that was used in a previous study examining vaccine acceptance among healthcare workers [62]. We also asked participants how many doses of a COVID-19 vaccine they had received. The third section also comprised questions about socio-demographic data — region of residency, age, ethnicity, gender, educational attainment, and whether or not they had received any vaccines before.

Prior to the launch of the DCE, a pilot was conducted with the research team to ensure comprehension and clarity of the questionnaire wording as well as the relevance of the attributes and levels. The questionnaire was then further refined using an iterative review process.

### Participant recruitment

This DCE was hosted on the 1000minds online platform and was live from 17 December 2021 to 7 March 2022. Participants aged 18 years or over and residing in New Zealand were eligible for inclusion. Participants were recruited from the research teams’ personal and professional networks, the University of Auckland Faculty of Medicine and Health Sciences research mailing list, Facebook advertisement, and NZ media (online and radio) via a press release. The DCE advertisement was also shared on Facebook pages with a high anti-vaccination following to capture those with strong anti-vaccination beliefs. This study was approved by the Auckland Health Research Ethics Committee (AH23637).

### Data quality checks

Internal validity of the DCE was assessed by the inclusion of a consistency check and identification of straight-lining– a phenomenon were participants consistently choose either the right or left-sided response [65]. The consistency check was placed at the end of the DCE and involved repeating two questions that the participants had already answered previously. Participants who failed to provide the same answers they had done previously for the consistency checks or straight-lined the questions had their data excluded from the final analysis. Additionally, participants who did not complete the DCE or repeatedly clicked the “They are equal” button also had their data excluded.

### Data analysis

Descriptive statistics were used to summarise the sample characteristics. The 1000minds online software was used to conduct the DCE analyses. In the DCE, the hypothetical vaccine was conceptualised as a particular combination of levels on the attributes, each representing different vaccines. Part-worth utilities quantify how much individuals prefer or value each level of an attribute. These values are based on the participant’s answers to the choice sets from the DCE so the part-worth utilities are consistent with the participant’s choices. The responses of the participants to each of the choice sets (i.e. the trade-off questions) are explicit pairwise rankings of all possible vaccine options based on two of the attributes. The software for the PAPRIKA method then uses linear programming techniques to derive weights called ‘part-worth utilities’ for the levels on each attribute, so that the preferences are weighted. These weights of preference are calculated for each level of every attribute based on the following:

the values for the highest levels across all the vaccine attributes sum to one, so each of these values represents the attribute’s relative weight overall. The lowest level of each attribute is equal to zero. The values of the levels in the middle depends on both the effect of the level’s middle position within the particular attribute as well as the attribute’s relative weight. The linear program includes a system of equalities or inequalities corresponding to the person’s answers to the trade-off questions that is solved simultaneously to obtain the utilities [54]. The measure of the preference a participant has for a particular vaccine is referred to as ‘utility’. The utilities indicate how much each level contributes to the overall desirability of an option. The higher the value, the higher preference. This is assumed to be additive across the attributes. This method also generates utilities for each individual. The ability to estimate individual-level data is useful as it allows the heterogeneity of people’s preferences about vaccines to be investigated.

A two-tailed unpaired t-test was used to compare the part-worth utilities for the thirteen attributes between participants scoring high versus low on the VCBS and between those who had not receive any doses of the COVID-19 vaccine versus those who had received one or more doses. Given that multiple tests were performed, we implemented the Bonferroni correction, so *p*-values less than 0.004 being considered significant.

## Results

### Study population

In total, 1432 people opened the DCE survey link, however, only 614 (42.9%) finished to completion. Of those that did not complete, 211 (14.7%) opened the link but did not start the DCE, 597 (41.6%) started but did not finish, and 10 (1%) were excluded as they either failed the consistency check or they repeatedly answered, ‘They are equal’. Additionally, a further three had to be excluded from the 614 respondents that completed the DCE, as they were not from New Zealand. This left 611 (42.7%) respondents available for the final analysis.

Table 2 shows the characteristics of the 611 respondents. On average, participants answered a mean (SD) of 28.4 (4.8) trade-offs, ranging from a minimum of 12 to a maximum of 35 trade-off scenarios. The most common number of trade-off was 32, answered by 92 participants. The median time to complete the survey was 11 min. All 15 regions of New Zealand were represented in the responses, with most (41.9%) from the Auckland region. The mean (SD) age was 45.9 (14.7), and most identified as female (70.9%), of European ethnicity (82.2%), and nearly half were university graduates (49.8%). Almost all reported having received a vaccine of any kind before (99.2%), and most respondents have had ≥ 2 doses of a COVID-19 vaccine (85.8%). The mean VCBS score (SD) was 18.5 (12.4) with the median score being 13.

**Table 2:** Characteristics of respondents (*n* = 611)

	N	%
Age		
18–24	53	8.7
25–44	237	38.8
45–64	240	39.3
65+	81	13.3
Region		
Auckland	256	41.9
Bay of Plenty	83	13.6
Wellington	74	12.1
Canterbury	56	9.2
Waikato	29	4.7
Other regions*	112	18.3
Gender		
Female	433	70.9
Male	161	26.4
Gender diverse	8	1.3
Prefer not to say	9	1.5
Ethnicity		
European	502	82.2
Asian	48	7.9
Māori	37	6.1
Middle Eastern/Latin American/African	11	1.8
Pacific Peoples	1	0.2
Other	12	2.0
Education		
High School	61	10
University/polytechnic	304	49.8
Post-graduate	238	39
Prefer not to say	8	1.3
Previous vaccine (any)		
Yes	606	99.2
No	4	0.7
Prefer not to say	1	0.2
COVID vaccine doses taken		
0	72	11.8
1	15	2.5
2	217	35.5
3	307	50.2
VCBS score		
Low (< 19)	393	64.3
High (≥ 19)	218	35.7

Other regions with less than 5% respondents individually: Hawke’s Bay, Manawatu-Wanganui, Nelson-Tasman, Northland, Otago, Southland, Taranaki

### DCE results– importance of vaccine attributes and ranking

Respondents answered a mean (SD) of 28.4 (4.8) trade-off questions. For the attributes where respondents had to self-rank attribute levels, most respondents ranked community-based healthcare (84.3%) as their first preferred place of administration, over workplaces and schools (10.1%), and hospitals (5.6%). For place of origin of vaccines, respondents preferred vaccines to be developed through a collaboration of multiple countries (43.5%) or in New Zealand (27.5%), with others preferring Europe (17.5), USA (10.5%) or Asia (1.0%). For route of administration, most rated intramuscular injections (48.3%) or oral delivery (39.4%) as their preferred delivery route, with some preferring skin patches (7.9%) or nasal sprays (4.4%).

**Fig. 3:**
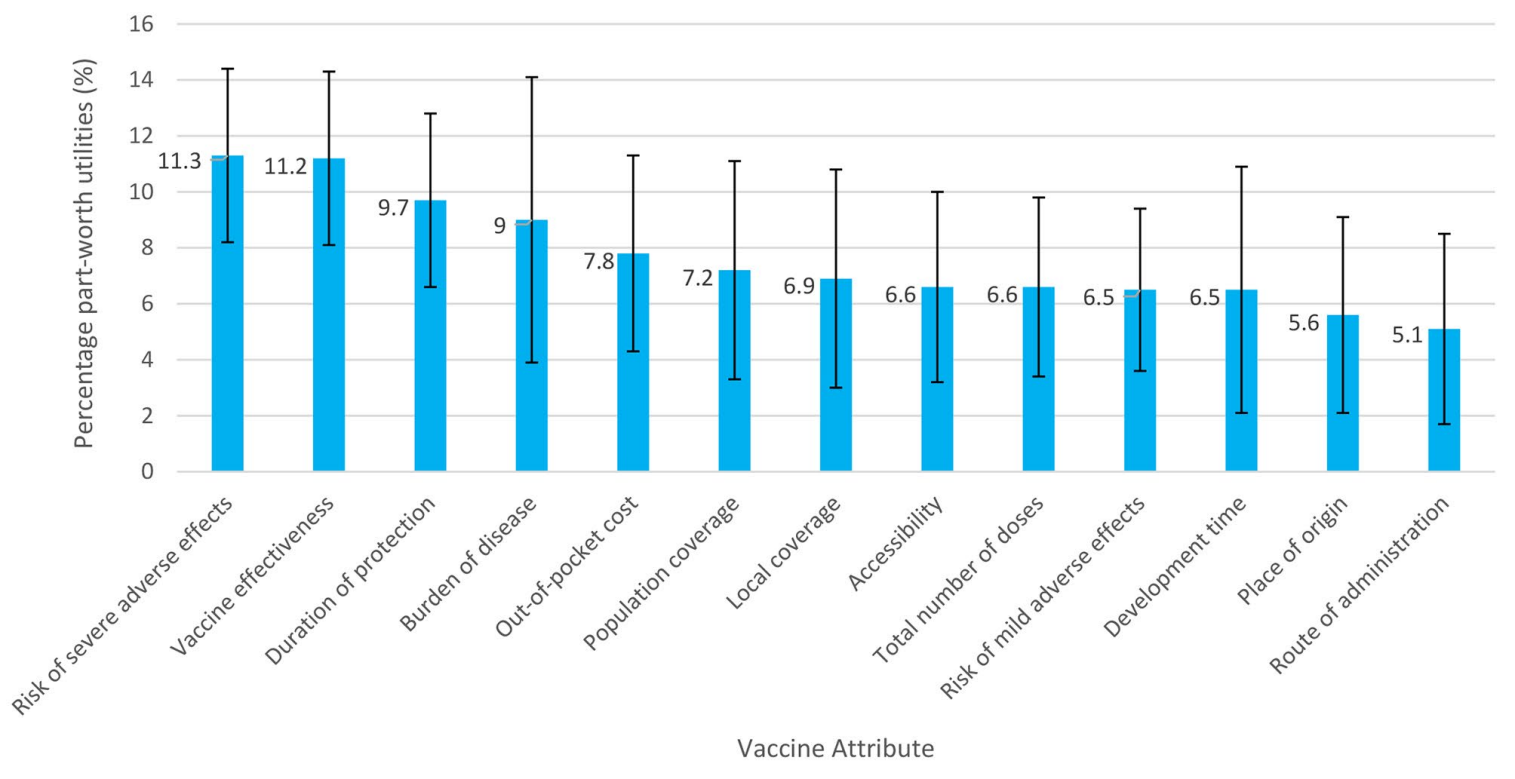
Mean percentage part-worth utilities by vaccine attribute* *n* = 611. *Higher percentage part-worth utilities indicate higher importance / ranking by respondents for that attribute

Figure 3 shows the percentage part-worth utilities for each vaccine attribute– the higher the percentage, the greater the importance of the attribute for respondents. Risk of severe adverse effects was the most highly valued attribute, with a mean (SD) part-worth utility of 11.3 (3.1)% followed by vaccine effectiveness (11.2 (3.1)%) and duration of protection (9.7 (3.1)%). The origin of the vaccine (5.6 (3.5)%) and its route of administration (3.4 (3.5)%) were ranked as the least important attributes.

### Differences in vaccine attribute preferences by vaccine conspiracy beliefs

With the VCBS, significant differences were found between high (≥ 19) and low scoring groups (< 19). Respondents that scored highly on the VCBS placed less value on the effectiveness of vaccines (9.4 (3.4)%) relative to low scorers (12.2 (2.4)%; *p* < 0.001). Additionally, high scorers were more likely to place greater value on the development time and the total number of doses, with mean part-worth utilities of 9.7 (4.4)% and 8.3 (3.5)%, respectively. This is in comparison to the mean part-worth utilities of low scorers: 4.8 (3.4)% and 5.7 (2.6)% (*p* < 0.001).

### Differences in vaccine attribute preferences by COVID-19 vaccination status

Respondents who had not received any COVID-19 vaccine doses ranked longer development times more highly (11.3 (3.6)% versus 5.9 (4.1)%, *p* < 0.001) and total number of doses required more highly (9.3 (3.3)% versus 6.2 (3.0)%, *p* < 0.001), than those who received one or more doses. In contrast, vaccine effectiveness (7.8 ( 3.1)% versus (11.7 (2.8)%, *p* < 0.001) and population coverage (4.3 (2.4)%) versus (7.6 (3.9)%, *p* < 0.001) were ranked lower by those who did not receive any doses of the COVID-19 vaccine than those who had received at least one dose.

## Discussion

This study is the first to explore the preferences of the New Zealand public with regards to different vaccine attributes [28]. We found that risk of severe adverse effects, vaccine effectiveness, and duration of protection were the three most valued attributes by the New Zealand public when it comes to vaccine decision-making. We also found that vaccine attributes such as the development time, the place of origin, and route of administration had the least influences on vaccine decision-making. The results highlight key important areas that health communications in New Zealand can focus on when disseminating information about vaccines to promote vaccine uptake.

These findings are in line with the results reported by other DCE studies, which have reported severe adverse effects and vaccine effectiveness as key influencing factors for vaccination. Gong et al. found that the risk of severe adverse effects was the strongest influencing factor for Chinese parents when deciding to vaccinate their children [46]. Dong et al. conducted a DCE with the Chinese public and found that the most preferred attribute for COVID-19 vaccines was effectiveness [35], similar to a United States study that reported vaccine effectiveness as the most important attribute for HIV vaccines [66]. In contrast, an Australian DCE found that, amongst adolescents, the most influential attribute for a hypothetical vaccine was the burden of disease [36], which was only ranked fourth in our DCE. The differences in age group may account for the differences in rankings as our DCE respondents had a higher median age whereas the Australian DCE focused on adolescent preferences. These findings from our study have important implications when considering vaccine promotion interventions. For clinical practice, healthcare workers may use these attribute rankings to encourage vaccine uptake by ensuring information is given that addresses a vaccine’s risk of severe adverse effects and highlights its effectiveness at preventing disease. A similar approach could be applied at a wider scale with nationwide vaccination campaigns such as for COVID-19 focusing on delivering information about severe adverse effects, vaccine effectiveness and duration of protection. Policymakers may incorporate these findings into guidelines to support immunisation and individuals’ decision-making processes.

Interestingly, when considering information about adverse effects, our study found risk of mild adverse effects was ranked relatively low in our sample, being rated as less important than attributes such as population coverage and the total number of doses. Other DCE studies have previously reported risk of mild adverse effects as an important influencing factor. Cameron et al. conducted a study in groups at risk of HIV infections and found that absence of mild adverse effects could significantly increase the probability of HIV vaccine uptake in Thailand [49]. However, their study did not include severe adverse effects as an attribute level and did not compare different risk probabilities, only presence or absence of minor adverse effects [49]. A Dutch DCE on influenza vaccines found that risk of mild adverse effects had an impact on vaccine uptake, though this was not the most important attribute [45]. Our differences in findings could be potentially explained by our study being undertaken during one of the peaks of the COVID-19 pandemic, where vaccine communication nationwide had focused on reassurance of the public that mild adverse reactions — such as muscle pain, redness, fever — were to be expected from vaccinations. As such, the NZ public may not have ranked mild adverse reactions highly as they may have felt they had received sufficient information about this, and that other attributes such as population coverage and total number of doses were more important deciding factors. Further qualitative research in the NZ public can help understand the reasons driving the rankings observed, and conducting a follow-up survey during a period when pandemic awareness is low. It is possible that individual preferences vary both within and between individuals, and over time, so conducting longitudinal DCE studies to explore how preferences change over time would be a useful area for future research.

Even though these results are useful for highlighting which vaccine attributes are considered most important at a population level, there was high heterogeneity between individuals within our study population, as the responses differed significantly [67]. Our study used the PAPRIKA DCE method, which generates utilities for each individual. This information has potentially useful clinical applications by making predictions about how the individuals themselves and groups will behave when it comes to informing decisions about vaccination. For an individual, the literature shows that the most effective medicines information is likely one that is personalised to the individual’s preferences [20]. Because the PAPRIKA methodology generates information about an individual’s preferences, there is potential for the DCE survey to be used in this way, where individuals can be invited to complete the DCE to generate their personal ranked list of vaccine attributes for health providers to use for tailoring information about the vaccine. However, the limitation of this approach is that it does not take population preference heterogeneity into account, which other studies that use logistic regression models do [35, 45, 46]. Our study sample was recruited via primarily through research networks and social media, and is unlikely to be representative of the views of the New Zealand population. The sample had a high percentage of females and with under-representation of Māori and Pacific communities. There was also a high dropout rate which is common to many DCEs due to the number of trade-offs and unfamiliarity with the DCE design. This could lead to a bias in the results, as the people who completed the study were likely the most motivated participants. As such, a self-selection and non-response bias may be present, and our results may not be generalisable to the wider New Zealand public. Our study was also conducted at the height of the COVID-19 pandemic which may have affected participants’ rankings. Despite our study questions being based on a hypothetical general vaccine, participants may have interpreted the question with only the COVID-19 vaccine in mind. A repeat of this DCE under different circumstances may generate different vaccine preferences. Future research should aim to recruit from populations that were not well represented in this DCE sample — such as rural populations and vaccine hesitant individuals — and utilise a non-online method to capture people that do not routinely use social media or partake in online studies.

Our study found that preferences appear to differ between people with a tendency towards strong conspiracy beliefs versus those with weaker beliefs, and between people who are and are not vaccinated against COVID-19. Whilst our sample did not have sufficient numbers to explore how different participant characteristics may affect attribute rankings, due to the small numbers within each participant group, our study findings provide an important foundation for future research to build on. The finding that rankings did differ significantly between those who had vaccinated against COVID-19 and those who had not, suggests that the rankings are associated with people’s decision to vaccinate or not and that these rankings may be influenced by their tendency towards conspiracy theories. A longitudinal follow-up study to see whether participant rankings can predict whether or not they vaccinate in the future would be useful to understand the association between vaccine preferences and vaccination.

## Conclusion

This study sought to investigate the vaccine preferences of the New Zealand public, by exploring the trade-offs that people were willing to make for different vaccine characteristics. Our results showed that risk of severe adverse effects, vaccine effectiveness, and duration of protection were ranked as the most important attributes. These rankings of attributes differed between groups with stronger versus weaker conspiratorial beliefs and between those who had received the COVID-19 vaccine compared to those who had not. Our findings could be beneficial to health care workers, policymakers, and vaccine manufacturers when deciding on what attributes to prioritise when developing, introducing, and promoting future vaccines to the public.

### Electronic supplementary material

Below is the link to the electronic supplementary material.

Supplementary Material 1

## Data Availability

The datasets used and/or analysed during the current study available from the corresponding author on reasonable request.
