# Supplementary material for: Vaccine decision making in New Zealand: a discrete choice experiment
Source: BMC Public Health. 2024 Feb 12;24:447. doi: 10.1186/s12889-024-17865-8 (PMC10863187; doi:10.1186/s12889-024-17865-8)
Supplement: Supplementary file 1 — Supplementary Material 1 [file 12889_2024_17865_MOESM1_ESM.docx]

**Supplementary material**

**Appendix A1: Literature review search terms used to inform DCE attributes and levels**

| **Topic** | **Search terms** | **Filters** |
| --- | --- | --- |
| Vaccines | vaccin* or immunis* or immuniz* | English language  Humans |
| Patient preferences | prefer* or “patient preference” or choice* or engage* or uptake or decision*”or willingness* or attitude* or perception* |  |

**Appendix A2: Summary of iterative changes made to DCE attributes and levels**

| Attribute | | | Description of attribute for respondents | | | | Levels | | | | Comments | |
| --- | --- | --- | --- | --- | --- | --- | --- | --- | --- | --- | --- | --- |
| Version 1 | Version 2 | Version 3 | Version 1 | Version 2 | Version 3 | Version 1 | | Version 2 | Version 3 |  | |  |
| Vaccine effectiveness | No change | No change | No description used | How effective the vaccine is at protecting against infection | The vaccine prevents disease in this proportion of people: | >80%  >70% | | 50%  70%  90% | No change | Original attribute retained from Version 1 | |  |
| Duration of protection | No change | No change | No description used | How long the vaccine protects you against infection | The vaccine protects you against infection for: | 1 year  5 years | | 1 year  10 years  Lifetime | No change | Original attribute retained from Version 1 | |  |
| Long-term adverse effects | Risk of mild adverse effects | No change | No description used | Whether or not you will get long term side effects | The chance of you having mild side effects (e.g. chills, fever, muscle aches) is: | Yes  No | | 0%  5%  10% | 1 in 50  1 in 20  1 in 10 | Attribute changed to mild adverse effects in Version 2 | |  |
| Short-term adverse effects | Risk of severe adverse effects | No change | No description used | Whether or not you will get short term side effects that go away in 2-3 days | The chance of you having severe side effects (e.g. needing to go to hospital) is: | Yes  No | | 0.0001%  0.001%  0.01% | 1 in 1,000,000 (million)  1 in 100,000  1 in 1,000 | Attribute changed to severe adverse effects in Version 2 | |  |
| Number of injections | Total number of doses | No change | No description used | How many doses you need to take | The number of doses you need to take for maximal protection: | One-off  Two doses  Three doses | | One  Two  Three | No change | Original attribute retained from Version 1 | |  |
| Country of origin | Place of origin | No change | No description used | Which country the vaccine originated from | The vaccine was developed in: | New Zealand  Asia  Europe  USA  Other | | New Zealand  Asia  Europe  USA  Multicountry | New Zealand  Asia  Europe  USA  Multiple countries | Original attribute retained from Version 1 | |  |
| Route of administration | No change | No change | No description used | How the vaccine is given | The vaccine is given by: | Injection into muscle  Oral (by mouth) | | Intramuscular injection  Skin patch  Oral (by mouth)  Nasal spray | Needle injection into muscle  Skin patch  Oral (by mouth)  Nasal spray | Original attribute retained from Version 1 | |  |
| Time taken to develop | Development time | No change | No description used | Time taken to develop the vaccine | How much time was spent on developing the vaccine: | 1 year  5 years  10+ years | | 1 year  5 years  10+ years | No change | Original attribute retained from Version 1 | |  |
| N/A (Attribute Added from Version 2) | Frequency of injections | Removed from Version 3 | N/A | How long you have to repeat doses | N/A | N/A | | Never – one-off only  Repeat every year  Repeat every 5 years | N/A | New attribute added from Version 2 but removed in Version 3 as felt this was captured in number of total doses required | |  |
| N/A (Attribute Added from Version 2) | Vaccine activation period | Removed from Version 3 | N/A | The vaccine starts protecting you in: | N/A | N/A | | 2 weeks  1 month  ≥3 months | N/A | New attribute added from Version 2 but removed in Version 3 as the timing of protection varies widely between individuals and vaccines | |  |
| N/A (Attribute Added from Version 3) | N/A (Attribute Added from Version 3) | Out-of-pocket cost | N/A | N/A | Per dose, the vaccine would cost you: | N/A | | N/A | $0  $5  $50  $100 - $150 | New attribute added from Version 3 to capture NZ context of subsidised vaccines versus out-of-pocket costs | |  |
| N/A (Attribute Added from Version 3) | N/A (Attribute Added from Version 3) | Burden of disease | N/A | N/A | The disease being prevented by the vaccine is: | N/A | | N/A | Common with mild symptoms. Hospitalisations are rare and the disease is not life-threatening.  Common with severe symptoms. Hospitalisations are common and the disease is life-threatening.  Rare with mild symptoms. Hospitalisations are rare and the disease is not life-threatening.  Rare with severe symptoms. Hospitalisations are common and the disease is life-threatening. | New attribute added from Version 3 as felt burden of disease not captured elsewhere in other attributes | |  |
| N/A (Attribute Added from Version 3) | N/A (Attribute Added from Version 3) | Accessibility | N/A | N/A | You can receive the vaccine in: | N/A | | N/A | Community care (e.g. GPs, pharmacies, community centres)  Workplaces and schools  Hospitals | New attribute added from Version 3 to reflect the New Zealand health environment of being able to access the vaccine from different settings  Final version had “Community care” changed to “Community-based healthcare”. “Drive-in vaccine centres” also added as an example of community-based healthcare. | |  |
| N/A (Attribute Added from Version 3) | N/A (Attribute Added from Version 3) | Local coverage | N/A | N/A | The proportion of your family and friends who are already vaccinated is: | N/A | | N/A | 20% of your family and friends are already vaccinated  50% of your family and friends are already vaccinated  80% of your family and friends are already vaccinated | New attribute added from Version 3 to capture influence of social norms | |  |
| N/A (Attribute Added from Version 3) | N/A (Attribute Added from Version 3) | Population coverage | N/A | N/A | The proportion of the population who are already vaccinated is: | N/A | | N/A | 20% of the population is already vaccinated  50% of the population is already vaccinated  80% of the population is already vaccinated | New attribute added from Version 3 to capture influence of social norms | |  |
